# Randomized Controlled Trials for Post-COVID-19 Conditions: A Systematic Review

**DOI:** 10.7759/cureus.67603

**Published:** 2024-08-23

**Authors:** Shastri Motilal, Rebecca Rampersad, Mercédes Adams, Sarah Goon Lun, Adesh Ramdhanie, Tricia Ruiz, Amresh Shah, Arien Wilkinson, Jadon Lewis

**Affiliations:** 1 Paraclinical Sciences Department, The University of the West Indies, St. Augustine Campus, Faculty of Medical Sciences, St. Augustine, TTO

**Keywords:** sars-cov-2 infection, covid-19, randomized controlled trials, systematic review, post-acute sequelae of covid-19

## Abstract

Post-coronavirus disease 2019 (COVID-19) syndrome or condition (PCS) is defined as new onset symptoms for at least three months following COVID-19 infection that has persisted for at least two months. Given the global sequelae of COVID-19, there is an urgent need for effective PCS interventions. The aim of this study was to systematically review all interventions for PCS tested in randomized controlled trials. In this International Prospective Register of Systematic Reviews (PROSPERO) registered (CRD42023415835) systematic review, PubMed, Google Scholar, and ClinicalTrials.gov databases were searched between 1st January 2020 and 30th April 2023. Inclusion criteria were (1) randomized controlled trials that tested interventions for (2) PCS as defined above. Studies were independently reviewed, and final decisions regarding extracted data and risk of bias were made by consensus. Trial findings were summarized qualitatively. The review included 23 trials with 1,916 subjects (mean age 44.9, 25.8% males) from 10 countries. The predominant symptom or function targeted by the interventions were general long COVID-19 symptoms (35%), fatigue (30%), breathlessness (17%), olfactory (17%), and brain function (9%). Overall, the majority of trials (74%) were at high risk of bias. A range of interventions were identified, including physical therapies, dietary and regenerative treatments, electrical stimulation, and digital wellness programs with variable effects. While a diverse range of interventions for PCS have been tested, their effectiveness varies, with threats to validity in most studies. Trials focusing on PCS mental health disorders, musculoskeletal complaints, and children are needed. Well-designed RCTs are needed to establish definitive interventions for PCS.

## Introduction and background

The RNA virus known as severe acute respiratory syndrome coronavirus 2 (SARS-CoV-2), which has ravaged the globe for the past three years, is the source of coronavirus disease 2019 (COVID-19). Due to mutations, there are newly emerging strains of COVID-19 that are highly transmissible without increased disease severity, which allows the virus to be a continuous threat to global health [1]. While a high percentage of COVID-19 patients have a full recovery after the initial illness, 20% experience mid- and long-term effects [2]. This is referred to as a "long COVID”, “post-COVID-19 condition” or "post-acute COVID-19 syndrome (PACS) [3].

PACS can persist for weeks, months, or longer (mid- or long-term), and while it can affect persons who had mild or no symptoms of the illness, it has a high prevalence in persons with a history of severe COVID-19 illness [3]. There are general, digestive, respiratory, cardiac, and neurological symptoms that epidemiological studies have found associated with PCS, some of which are fatigue, post-exertional malaise, fever, stomach pain, diarrhea, cough, difficulty breathing, headaches, depression, and joint or muscle pain [3].

Despite the abundance of systematic reviews and clinical trials on acute COVID-19 treatment, an August 2022 review found only two trials specifically addressing interventions for PCAS [2]. With the emergence of new interventional studies for PCAS, an updated review is warranted. The aim of this research was to systematically review all interventional studies on PCAS, assess their quality, and synthesize their findings.

## Review

Methods

Search Strategy and Selection Criteria

This systematic review included randomized controlled trials (RCTs), including cross-over trials, that examined interventions on subjects with post-COVID-19 conditions. This review sought all RCTs that examined interventions on subjects with PCAS in keeping with the WHO definition [4]. Participants in eligible trials had continuing or newly developed symptoms at least three months following initial SARS-CoV-2 infection, with persistent symptoms at trial entry without any other explanation. Trials with symptomatic participants at less than 90 days between acute infection and enrolment were excluded. Studies were included if they were published in English from the period 01/01/2020 to 30/04/2023. Non-eligible studies were animal studies, cohort studies, case-control studies, in-vitro studies, and trials without a control group.

The literature search was conducted using PubMed, the clinicaltrial.gov database, and Google Scholar for grey literature. Creation of title listing for Google Scholar searches was done by Publish or Perish Software which was extracted into spreadsheets to screen titles [5]. Authors of studies still recruiting or with unclear recruitment status were contacted for any available preliminary published data. The search strategy used terms related to the post-COVID-19 condition. Full details of the search syntax can be found in Table 1.

**Table 1:** Search strategy

Database searched	Search Syntax
Pubmed	("post acute covid 19 syndrome"[Supplementary Concept] OR "post acute covid 19 syndrome"[All Fields] OR "post acute covid syndrome"[All Fields]) AND ("clinical trials as topic"[MeSH Terms] OR ("clinical"[All Fields] AND "trials"[All Fields] AND "topic"[All Fields]) OR "clinical trials as topic"[All Fields] OR "trial"[All Fields] OR "trial s"[All Fields] OR "trialed"[All Fields] OR "trialing"[All Fields] OR "trials"[All Fields])
Google Scholar Search Syntax	"randomized controlled trial" OR "randomised controlled trial" AND "post COVID-19 syndrome" OR "post COVID-19 condition" OR "post COVID-19 condition" OR "post-acute sequelae of SARS CoV 2 infection" OR "Long COVID-19" OR “Long COVID-19”
ClinicalTrials.gov Search Terms (URL format)	https://clinicaltrials.gov/ct2/results?term=randomized+controlled+trial&cond=%28%22post-covid-19+syndrome%22+OR+%22post-acute+sequelae+of+SARS-CoV-2+infection%22+OR+%22PASC%22+OR+%22long+covid%22+OR+%22long-haul+covid%22%29&age_v=&gndr=&type=Intr&rslt=&Search=Apply

Study Quality and Data Analysis

Paired reviewers (JL, AW; AS, TR; RR, SGL; and MA, AR.) independently screened articles by title, abstract, and then full text to determine eligibility for final inclusion. Any disagreements between the authors were discussed, and a final decision was made by SM after consensus meetings with reviewers. SM extracted data from the final selection of articles into a spreadsheet, which was checked by a second reviewer JL. Missing data was requested from the study authors firstly by SM and then JL if no initial response. No assumptions were made where data was missing.

Using the most recent version of the Cochrane risk-of-bias (RoB2) tool, two authors independently assessed the risk of bias for each study’s primary outcome [6]. For the deviation from intended interventions RoB2 domain, the effect of assignment to intervention was assessed when intention-to-treat was reported. For studies that did not use an intention-to-treat approach, the effect of adherence to treatment was assessed and presented for this RoB2 domain. For trials that were crossover by design, the supplemental version of RoB2 tailored for assessing such designs was used [7]. Disagreements were resolved by consensus.

A qualitative synthesis of all studies was completed. The main results for the primary outcome from each trial were reported comparatively. Risk of bias measures were reported as percentages by domain and qualitatively by trial. The proposal for this review was registered in the International Prospective Register of Systematic Reviews (PROSPERO) database (No. CRD42023415835) and adhered to the Preferred Reporting Items for Systematic Reviews and Meta-Analyses (PRISMA) standards as shown in Table 2 [8,9].

**Table 2:** PRISMA Checklist N/A: Not applicable; PRISMA: Preferred Reporting Items for Systematic Reviews and Meta-Analyses

Section and Topic	Item #	Checklist item	Section where the item is reported
Title	1	Identify the report as a systematic review.	Title
ABSTRACT			
Abstract	2	See the PRISMA 2020 for Abstracts checklist.	Abstract
INTRODUCTION			
Rationale	3	Describe the rationale for the review in the context of existing knowledge.	Background
Objectives	4	Provide an explicit statement of the objective(s) or question(s) the review addresses.	Background
METHODS			
Eligibility criteria	5	Specify the inclusion and exclusion criteria for the review and how studies were grouped for the syntheses.	Methods
Information sources	6	Specify all databases, registers, websites, organisations, reference lists and other sources searched or consulted to identify studies. Specify the date when each source was last searched or consulted.	Methods
Search strategy	7	Present the full search strategies for all databases, registers and websites, including any filters and limits used.	Methods
Selection process	8	Specify the methods used to decide whether a study met the inclusion criteria of the review, including how many reviewers screened each record and each report retrieved, whether they worked independently, and if applicable, details of automation tools used in the process.	Methods
Data collection process	9	Specify the methods used to collect data from reports, including how many reviewers collected data from each report, whether they worked independently, any processes for obtaining or confirming data from study investigators, and if applicable, details of automation tools used in the process.	Methods
Data items	10a	List and define all outcomes for which data were sought. Specify whether all results that were compatible with each outcome domain in each study were sought (e.g. for all measures, time points, analyses), and if not, the methods used to decide which results to collect.	Methods
	10b	List and define all other variables for which data were sought (e.g. participant and intervention characteristics, funding sources). Describe any assumptions made about any missing or unclear information.	Methods
Study risk of bias assessment	11	Specify the methods used to assess risk of bias in the included studies, including details of the tool(s) used, how many reviewers assessed each study and whether they worked independently, and if applicable, details of automation tools used in the process.	Methods
Effect measures	12	Specify for each outcome the effect measure(s) (e.g. risk ratio, mean difference) used in the synthesis or presentation of results.	N/A
Synthesis methods	13a	Describe the processes used to decide which studies were eligible for each synthesis (e.g. tabulating the study intervention characteristics and comparing against the planned groups for each synthesis (item #5)).	Methods
	13b	Describe any methods required to prepare the data for presentation or synthesis, such as handling of missing summary statistics, or data conversions.	N/A
	13c	Describe any methods used to tabulate or visually display the results of individual studies and syntheses.	Methods
	13d	Describe any methods used to synthesize results and provide a rationale for the choice(s). If meta-analysis was performed, describe the model(s), method(s) to identify the presence and extent of statistical heterogeneity, and software package(s) used.	Methods. Only qualitative synthesis was possible
	13e	Describe any methods used to explore possible causes of heterogeneity among study results (e.g. subgroup analysis, meta-regression).	N/A
	13f	Describe any sensitivity analyses conducted to assess the robustness of the synthesized results.	N/A
Reporting bias assessment	14	Describe any methods used to assess the risk of bias due to missing results in a synthesis (arising from reporting biases).	Part of RoB2
Certainty assessment	15	Describe any methods used to assess certainty (or confidence) in the body of evidence for an outcome.	N/A
RESULTS			
Study selection	16a	Describe the results of the search and selection process, from the number of records identified in the search to the number of studies included in the review, ideally using a flow diagram.	Results
	16b	Cite studies that might appear to meet the inclusion criteria, but which were excluded, and explain why they were excluded.	Results
Study characteristics	17	Cite each included study and present its characteristics.	Results
Risk of bias in studies	18	Present assessments of risk of bias for each included study.	Results
Results of individual studies	19	For all outcomes, present, for each study: (a) summary statistics for each group (where appropriate) and (b) an effect estimate and its precision (e.g. confidence/credible interval), ideally using structured tables or plots.	N/A
Results of syntheses	20a	For each synthesis, briefly summarise the characteristics and risk of bias among contributing studies.	N/A
	20b	Present results of all statistical syntheses conducted. If meta-analysis was done, present for each the summary estimate and its precision (e.g. confidence/credible interval) and measures of statistical heterogeneity. If comparing groups, describe the direction of the effect.	N/A
	20c	Present results of all investigations of possible causes of heterogeneity among study results.	N/A
	20d	Present results of all sensitivity analyses conducted to assess the robustness of the synthesized results.	N/A
Reporting biases	21	Present assessments of risk of bias due to missing results (arising from reporting biases) for each synthesis assessed.	Results
Certainty of evidence	22	Present assessments of certainty (or confidence) in the body of evidence for each outcome assessed.	N/A
DISCUSSION			
Discussion	23a	Provide a general interpretation of the results in the context of other evidence.	Discussion
	23b	Discuss any limitations of the evidence included in the review.	Discussion
	23c	Discuss any limitations of the review processes used.	Discussion
	23d	Discuss implications of the results for practice, policy, and future research.	Discussion
OTHER INFORMATION			
Registration and protocol	24a	Provide registration information for the review, including register name and registration number, or state that the review was not registered.	Methods
	24b	Indicate where the review protocol can be accessed, or state that a protocol was not prepared.	Methods
	24c	Describe and explain any amendments to information provided at registration or in the protocol.	N/A
Support	25	Describe sources of financial or non-financial support for the review and the role of the funders or sponsors in the review.	Disclosures
Competing interests	26	Declare any competing interests of review authors.	Disclosures
Availability of data, code, and other materials	27	Report which of the following are publicly available and where they can be found: template data collection forms; data extracted from included studies; data used for all analyses; analytic code; any other materials used in the review.	Data availability statement

Results

**Figure 1:**
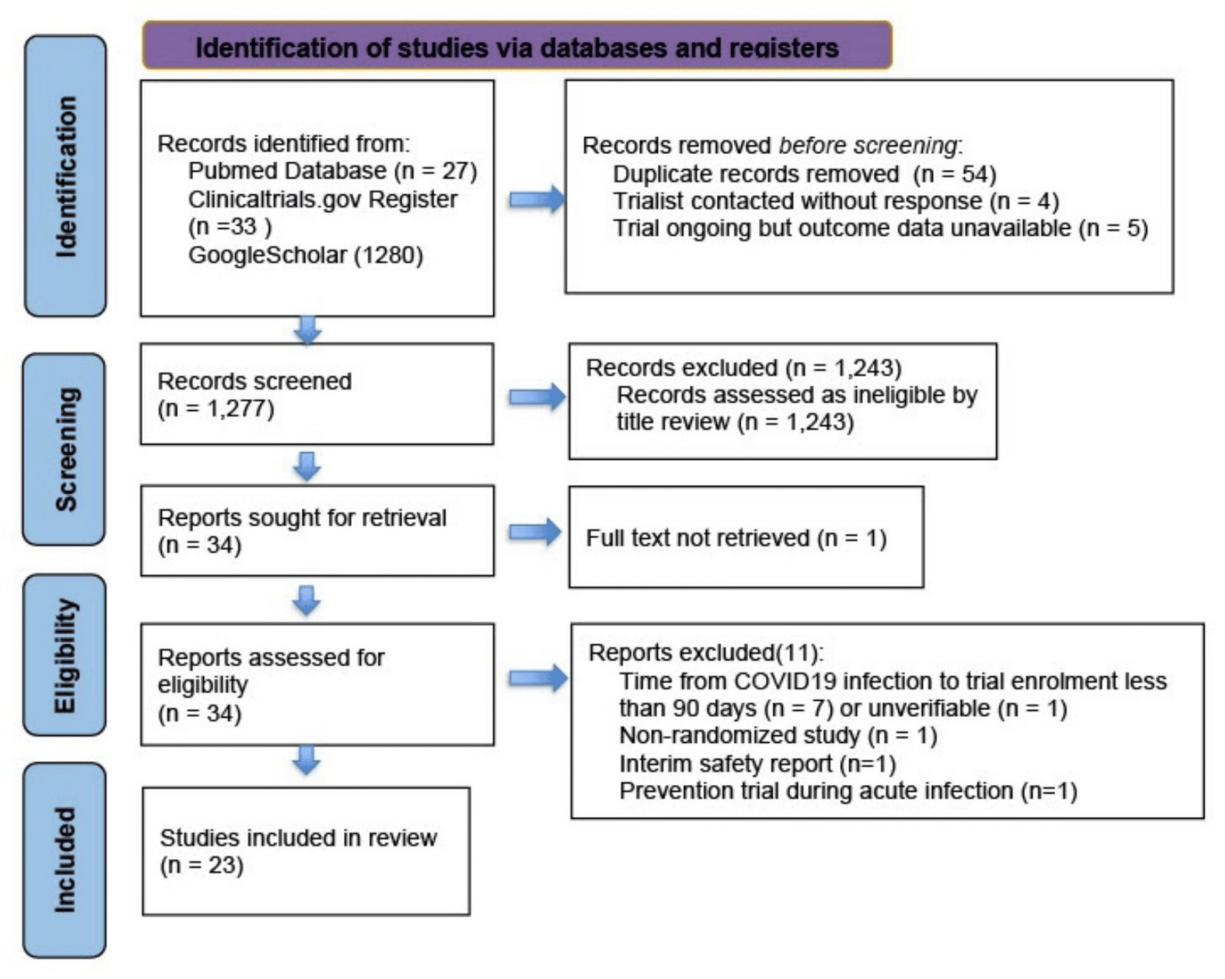
PRISMA diagram depicting the selection of eligible studies PRISMA: Preferred Reporting Items for Systematic Reviews and Meta-Analyses

From an initial search yielding 1,340 articles, a total of 23 trials were included in this review which were 18 original published papers, four preprints and one conference abstract. Figure 1 depicts the selection of studies for this review.

The excluded trials are detailed in Table 3.

**Table 3:** Excluded studies

Reasons for excluded studies
The duration of COVID-19 symptoms at the time of enrolment was less than 90 days [10-16].
The duration of COVID-19 symptoms was not reported and the author was unable to verify [17].
Interventional study but longitudinal (not randomized) [18].
Interim safety report [19].
Examined prevention of post-COVID19 condition in acutely infected participants [20].

Collectively, the included trials allocated 1,916 subjects from 10 countries (Brazil, China, Denmark, France, Israel, Italy, Poland, Spain, United Kingdom, and the USA). The weighted mean age of all subjects was 44.9 years with 25.8% males. There was one pediatric study whose participants had an average age of 10.8 years, and 42% were males. The majority of studies (83%) recruited participants from outpatient clinics or the community. Of the 17 studies that reported a mean time from initial COVID-19 infection to enrolment or receipt of intervention, the overall mean was 256.6 days. For the remaining six studies, the range of time between acute infection and enrolment spanned 90-360 days. The predominant symptom or function targeted by the interventions in the trials were general long-COVID-19 symptoms (35%), fatigue (30%), breathlessness (17%), olfactory dysfunction (17%), and brain function (9%). With regards to the diagnosis of COVID-19, 10 (43%) of the trials did not explicitly state the use of polymerase chain reaction (PCR), however, all these studies stated in their inclusion criteria that participants must have had prior COVID-19 infection diagnosed.

Sample sizes were calculated in the majority (78%) of studies. Of the 18 studies with calculated sample sizes, the trial was adequately powered by allocated subjects in 15 (83%). Blinding status was double, single, and unblinded in 10 (44%), six (26%), and 7 (30%) of the trials, respectively. Published protocols were available for three (13%) of the studies, with the majority of studies (83%) registered on clinicaltrials.gov. Study characteristics of the included trials are shown in Table 4 [21-43].

**Table 4:** Characteristics of included trials

Authors, year (location)		Main post-COVID-19 condition, symptom or function assessed	Age of entire sample years^*^		Proportion males		Time between COVID-19 diagnosis and enrolment or intervention. (days)^#^		Numbers of participants allocated, Intervention group(s) description		Numbers of participants allocated, Control group(s) description		Primary outcomes assessed	Primary outcomes main results
Badran et al., 2022 (USA) [21]		Long covid-19 symptoms	48.5		33%		213		n=6, two, one-hour transcutaneous auricular vagal nerve stimulation sessions per day, for a period of 4 weeks		n=6, sham transcutaneous auricular vagal nerve stimulation sessions per day, for a period of 4 weeks		Feasibility and safety of intervention	By the 5th of 6 sessions, no one in either group required help. No unanticipated adverse events occurred throughout the trial. There were 2 instances of mild skin irritation
Calvani et al., 2023 (Italy) [22]		Fatigue	47.9		35%		252.6		n=23, twice daily oral supplementation with a combination of 1.66g L-arginine and 500mg liposomal vitamin C for 28 days		n=23, placebo		Serum concentrations of l-arginine, citrulline, ornithine and other amino acids with ratios.	Serum l-arginine concentrations and l-arginine/ asymmetric dimethylarginine increased significantly compared with placebo.
Catalogna et al., 2022 (Israel) [23]		Brain connectivity	48.2 ± 10		39.20%		≥ 90		n=37, 40 sessions of Hyperbaric Oxygen Therapy		n=36, sham hyperbaric chamber treatment		Cognitive assessments evaluated by computerized cognitive testing battery	Hyperbaric Oxygen Therapy notably enhanced cognitive and executive functions, lessened psychological symptoms, and altered brain connectivity and fractional anisotropy in key brain regions (p<0.05)
Chung et al., 2022 (China) [24]		Olfactory dysfunction	44		N/A		157		n=10, short–course (14 days) oral Vitamin A (25,000 IU per day) in combination with aerosolisation diffuser olfactory training thrice daily (combination group); n=9, Olfactory training alone (standard care group)		n=5, clinical observation (control group) for 4 weeks.		Difference in measured olfactory function by the butanol threshold test (BTT) between baseline and end-of-treatment.	At end-of-treatment, mean butanol threshold test scores were significantly higher for the combination group when compared to control (p<0.001) and standard care (p=0.009) groups.
del Corral et al., 2023 (Spain) [25]		Fatigue and Dyspnea	46.4		28%		351		n=22, home-based inspiratory muscle training programme x 8 weeks, n=22, home-based inspiratory and expiratory muscle training programme x 8 weeks		n=22, sham home-based inspiratory muscle training programme x 8 weeks, n=22, sham home-based inspiratory and expiratory muscle training programme x 8 weeks		Health-related quality of life and exercise tolerance	At post-intervention, there was a statistically significant and large (d>0.90) improvement in quality of life, but not in exercise tolerance, in the inspiratory and expiratory muscle group compared with the sham equivalent.
Di Stadio et al., 2022 (Italy) [26]		Olfactory dysfunction and cogntive impairment	43.5 ± 14.6		35%		≥ 180		n=130, Daily treatment with ultramicronized palmitoylethanolamide and luteolin supplements, oral supplement and olfactory training.		n=55, Daily treatment with placebo and olfactory training.		Change over time in Threshold, Discrimination and Identification of olfactory dysfunction scores	Significant differences in olfactory scores were present at the 90-day experimental endpoint, (p <0.00001)
Hansen et al., 2023 (Denmark) [27]		Long COVID-19 symptoms	49		25%		289		n=59, oral capsules of Coenzyme Q10 in a dose of 500 mg/day or placebo for 6 weeks, with crossover treatment after a 4-week washout period.		n=60, placebo oral capsules for 6 weeks, with crossover treatment after a 4-week washout period.		Change in the number and/or severity of Post COVID19 Condition related symptoms and health index status	The difference between Coenzyme Q10 and placebo was not significant with respect to either the change in health index (p = 0.45) or the change in post-COVID related symptom score (p = 0.32)
Hawkins et al., 2022 (USA) [28]		Fatigue	19–49		0%		180-270		n=20, a blend of essential oils extracted from the following plants: thyme (Thymus vulgaris), orange peel (Citrus sinensis), clove bud (Eugenia caryophyllus), and frankincense (Boswellia carterii) for 2 weeks		n=20, placebo product contained an inert, odorless fractionated coconut oil		Total score on the Multidimensional Fatigue Symptom Inventory Short Form	Individuals who inhaled the essential oil blend for 2 weeks had significantly lower fatigue scores after controlling for baseline scores, employment status, BMI, olfactory function, and time since diagnosis, with a large effect size ( p = .020)
Jimeno-Almazán et al., 2022 (Spain) [29]		Long covid-19 symptoms	45.2 ± 9.5		26%		231		n=20, 8 weeks of a tailored and supervised multicomponent adapted exercise program for chronic obstructive pulmonary disease and cardiovascular disease		n=20,Informed (non-supervised) to follow the WHO guidelines: Support for Rehabilitation: Self-Management after COVID-19 Related Illness.		Health related quality of life by the 12-item Short Form Survey, calculating the mental component and physical component scores. Anxiety and depression symptoms, perception of dyspnea and the disability produced by this, Fatigue intensity, myalgic encephalomyelitis/chronic fatigue syndrome symptoms and functional limitations after COVID-19	The magnitude of the change pre–post intervention favored the exercise group in cardiovascular and strength markers (p < 0.05). In addition, exercise intervention resulted in a significantly better quality of life, less fatigue, less depression, and improved functional status, as well as in superior cardiovascular fitness and muscle strength compared to controls (p < 0.05).
Jimeno-Almazán et al., 2023 (Spain) [30]		Long COVID-19 symptoms	45.3 ± 8.0		31%		≥ 84		n=20, concurrent training; n=23, concurrent training with respiratory muscle training; n=17, respiratory muscle training all for 8 weeks		n=20, Control group followed WHO Self Management home-based program		Cardiorespiratory fitness and muscle strength	No significant changes in VO2 max, handgrip strength and bench press one repetition among all 4 groups. (P>0.05). Both concurrent training intervention groups significantly different to control group for bench press and half squat mean velocities, and half-squat one-repetition maximum (P<0.05)
Khan et al., 2023 (USA) [31]		Olfactory dysfunction	41		14%		180		n=61, bimodal (visual with olfactory) training with patient-preferred scents; n=61, bimodal (visual with olfactory) training with physician-assigned scents; n=58, unimodal training with patient-preferred scents; n=60, unimodal training with physician-assigned scents.		n=35, control		Olfactory assessment, Clinical Global Impressions Severity and Improvement, and Olfactory Dysfunction Outcomes Rating	The mean change in olfactory dysfunction score preintervention to postintervention was 11.6 points (95% CI, 9.2-13.9), which was not deemed clinically important nor significantly different between arms.
McNarry et al., 2022 (United Kingdom) [32]		Breathlessness	46.6 ±12.2		12%		270±126		n=176, three unsupervised inspiratory muscle training sessions/week, on non-consecutive days, for eight weeks		n=48, usual care		Health-related quality of life	There was no difference between groups in total score post-intervention. Muscle training elicited clinically meaningful improvements in the subdomains of breathlessness and chest symptoms (P<0.05)
Ogonowska-Slodownik et al., 2023 (Poland) [33]		Fatigue, breathlessness	10.8		42%		90-240		n=25, Aqua groups training sessions were conducted twice a week, 45 minutes per session, for eight weeks. n=23, Land groups training sessions were conducted twice a week, 45 minutes per session, for eight weeks.		n=26, no additional exercise to their normal routine		Exercise capacity, measured using the modified Balke treadmill protocol, and fatigue, measured using the Cumulative Fatigue Symptoms Questionnaire.	No significant correlations were found between total fatigue scores and VO2max, pre and post-intervention, in the groups
Oliver-Mas et al., 2023 (Spain) [34]		Fatigue	45.66		21%		620		n=23, Transcranial direct current stimulation consisting of eight sessions for eight days over the course of two consecutive weeks		n=24, sham transcranial stimulation		The Modified Fatigue Impact Scale score was used as the primary endpoint.	There were no statistically significant intergroup differences in the percentage of patients showing a clinically significant change in fatigue scores at the end of treatment (P = 0.440)
Palau et al., 2022 (Spain) [35]		Long COVID-19 symptoms	50.4 ± 12.2		58%		362		n=13, Inspiratory muscle training at home twice daily using a threshold inspiratory muscle trainer for 12 weeks		n=13, usual care		Average change from baseline in mean peak VO2	The mean of peakVO2 was 22.2 mL/kg/min (95% CI 21.3 to 23.2) compared to control, p<0.001
Philipe et al., 2022 (United Kingdom) [36]		Breathlessness	49 ± 12		17%		320		n=74, One-hour online sessions with the English National Opera Breathe Programme for six weeks		n=76, Usual care		Change in health-related quality of life using RAND 36-item short form survey instrument mental health composite and physical health composite scores	Mental health composite score change was statistically significant compared to placebo P =0.047. Physical health composite score change was not statistically significant compared to control P=0.54.
Samper-Pardo et al., 2023 (Spain) [37]		Long COVID-19 symptoms	48.3		20%		112		n=52, ReCoVery rehabilitative phone application over three months		n=48, current subject routines and refrain from beginning any rehabilitation or similar activities over three months		Quality of life measures physical and mental health	Adherence to the ReCoVery APP was low, and was not significantly more effective as compared to no intervention.
Santana et al., 2023 (Brazil) [38]		Fatigue	53		36%		90-360		n=35, High definition Transcranial direct current stimulation 10 sessions over five weeks plus standard rehabilitation		n=35, sham stimulation 10 sessions over five weeks plus standard rehabilitation		Fatigue severity as assessed by the Modified Fatigue Impact Scale	The active direct current stimulation group had a significantly greater reduction in fatigue (P < .001)
Tosato et al., 2022 (Italy) [39]		Fatigue	50.5 ± 16.5		35.00%		240		n=25, twice daily oral supplementation with a combination of 1.66g L-arginine and 500 mg liposomal vitamin C for 28 days		N=25, placebo		The change from baseline to day 28 in distance walked on a 6min walk test	l-arginine plus vitamin C significantly increased the distance walked on the 6 min walk test (p = 0.001)
Vallier et al., 2023 (France) [40]		Long COVID-19 symptoms	54.8 ± 16.0		71%		140.9		n=9, inpatient pulmonary rehabilitation group		n=8, home pulmonary rehabilitation group		Distance covered in the six-minute walk test	The distance covered in the shows significant improvements, between pre- and post-rehabilitation program in both groups, with no significant interaction between time and group (P=0.420)
Versace et al., 2022 (Italy) [41]		Cortical plasticity	49.9 ± 11.4		35.30%		296.7		n=17, palmitoylethanolamide/luteolin ( 700mg + 70mg) orally twice daily for 8 weeks.		n=17, sublingual inert microgranules orally twice daily for 8 weeks.		Intracortical GABA-ergic neurotransmission indexed with long-interval intracortical inhibition	long-interval intracortical inhibition between groups was statistically significant at P = 0.034
Yan et al., 2022 (USA) [42]		Olfactory dysfunction	44.1		50%		264		n=18, 1 mL of either platelet rich plasma injected submucosally into bilateral olfactory clefts under endoscopic visualization over 4 weeks period		n=12, 1 mL of sterile saline injected submucosally into bilateral olfactory clefts under endoscopic visualization over 4 weeks		Change in Sniffin’ Sticks score (threshold, discrimination, and identification) from baseline.	Active treatment resulted in a greater improvement (p = 0.047) in olfaction compared with the placebo group at 3 months and a higher response rate
Zilberman-Itskovich et al., 2022 (Israel) [43]		Long COVID-19 symptoms	48.1		40%		165.3		n=37, 40 daily sessions of hyperbaric oxygen therapy		n=36, sham hyperbaric chamber treatment		Cognitive assessment as evaluated by a computerized cognitive testing battery	There was a significant group-by-time interaction in the global cognitive score post-oxygen therapy compared to the control group (p= 0.038). Both attention and executive function domains had significant group-by-time interactions (p= 0.04 and p= 0.05 respectively)

**Figure 2:**
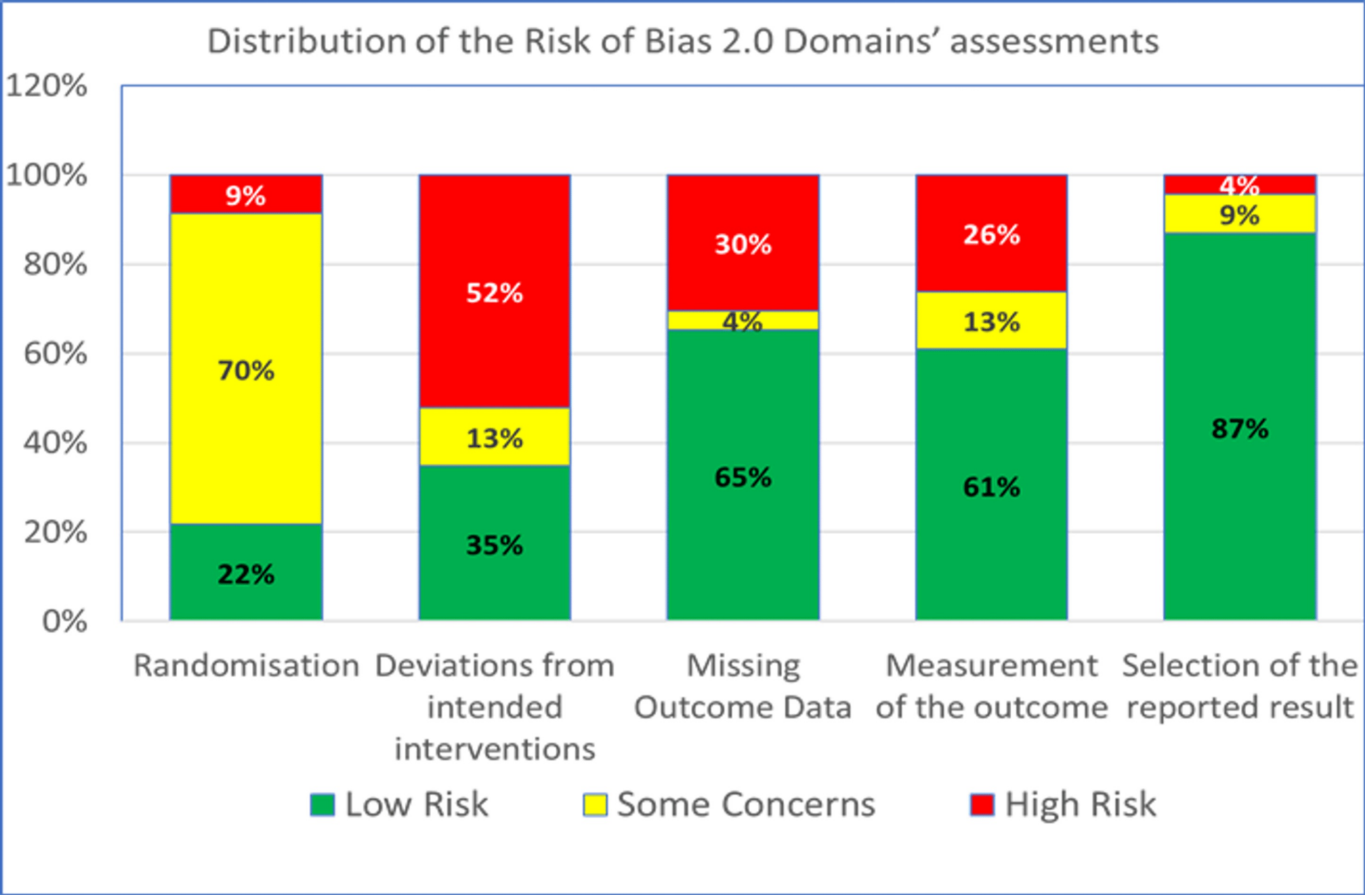
Distribution of risk of bias by study domain [21-43]

The risk of bias of the 23 included studies are shown by domain in Figure 2.

**Figure 3:**
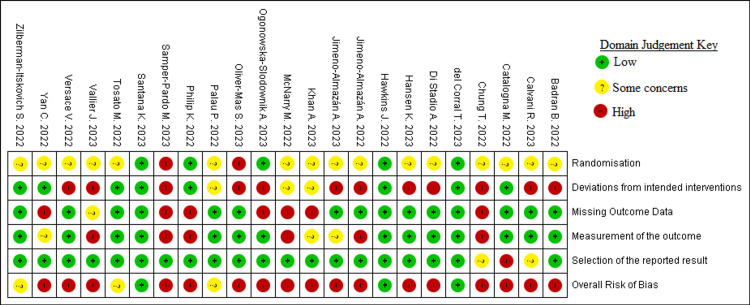
Risk of bias assessments for individual studies [21-43]

Overall, the majority of trials (74%) were at high risk of bias, with some concerns in three (13%) and low risk in three (13%) trials. The second RoB domain (deviation from intended interventions) was at greatest risk for high concerns. Almost a third (7/23) of the studies carried out an intention-to-treat (ITT) analysis, while the remainder did a per-protocol analysis. None of the studies that did a per-protocol analysis reported any evidence of appropriate analyses used to estimate the effect of adhering to the intervention. The proportion of ITT studies that had a high risk of bias on domain 2 was 0% vs. 75% of the per-protocol analysis studies (*P*=0.001). The proportion of ITT studies that had a high overall risk of bias was 43% vs. 88% of the per-protocol analysis studies (*P*=0.045) Blinding status (double-blind vs non-double-blind) was independent of overall bias risk (*P*=0.341). Figure 3 summarizes the judgment for each RoB domain and the overall risk for all studies.

The agreement on all five RoB domains after the preliminary independent review was 86%. A range of interventions were employed across the 23 trials, which encompassed physical therapies, dietary supplements, electrical stimulation, and digital wellness programs. The specifics of the treatments tested and their reported effects on the trials’ primary outcomes are summarized below by PCAS condition.

Eight trials tested different interventions for non-specific long COVID-19 symptoms. Interventions included a home-based training program using a threshold inspiratory muscle trainer [35], a rehabilitative phone app [37], transcutaneous auricular vagal nerve stimulation [21], hyperbaric oxygen therapy [43], high-dose Coenzyme Q10 [27], home-based and inpatient pulmonary rehabilitation [40], supervised therapeutic exercise [29], and concurrent training [30]. Of these, significant positive outcomes were reported for the home-based training program [30], vagal nerve stimulation [21], oxygen therapy [43], and supervised therapeutic exercise [29]. Conversely, the rehabilitative phone app, CoQ10, and both home-based and inpatient pulmonary rehabilitation didn't show significant comparative differences.

Seven studies focused on PCAS patients mainly suffering from fatigue. Interventions involved L-arginine and vitamin C supplementation [22,39], high-definition transcranial direct current stimulation [34,38], home-based respiratory muscle training [25], water-based and land-based exercise training programs [33] and essential oil inhalation [28]. Significant improvement was noted in patients undergoing L-arginine and Vitamin C supplementation [39], home-based respiratory muscle training [25], and essential oil inhalation [28]. There were mixed results in those who received high-definition transcranial direct current stimulation [34,38]. The other interventions did not show significant comparative benefits in fatigue scores.

Two trials separately investigated the effect of co-ultramicronized palmitoylethanolamide/luteolin [41] and hyperbaric oxygen therapy [23] on brain function. Both interventions led to significant enhancements in brain function parameters. Four studies investigated interventions for breathlessness PACS subjects, including an online breathing and wellbeing program [36], unsupervised inspiratory muscle training [32], a home-based respiratory muscle training program [25], and water and land-based exercise programs [33]. Significant results were seen for the online breathing program and inspiratory muscle training, improving mental health [36] and reducing breathlessness [32], respectively. The other two trials did not demonstrate significant improvements [25,33].

Four studies examined interventions for PCAS patients suffering from olfactory dysfunction. Three of the trials, which investigated oral ultramicronized palmitoylethanolamide and luteolin supplements [26], a combination of short-course oral vitamin A with aerosolization diffuser olfactory training [24], and platelet-rich plasma injections [42] found significant improvements. The study that examined bimodal visual-olfactory training with patient-preferred scents did not show any difference between the active intervention arms [31].

Discussion

From an initial search yielding 1,340 articles, a total of 23 randomized controlled trials were included in this systematic review. The various trial settings were geographically diverse, spanning the American, Asian and European continents with mostly outpatient participants. The gender predominance of women seen in the studies of this review is in keeping with the literature. In one cohort, long COVID-19 reportedly occurred more than three times the odds in women compared to men [44]. The studies found in this review enrolled predominantly adults with only one pediatric trial. The prevalence of PCAS in children has been estimated to be around 23%, almost half that of adults [45]. This may explain the paucity of PCAS trials in children and adolescents. Meta-analysis and Grading of Recommendations Assessment, Development and Evaluation (GRADE) assessments could not be done as part of this review as there were no studies assessing the same population, intervention, and outcomes. This, coupled with the high risk of bias in most trials, limits recommendations that can be made in favor of any of the interventions examined in this review.

Post-Acute Sequelae of Non-COVID-19 Viral Illnesses

Long haulers following viral illnesses are not specific to COVID-19. Symptoms similar to PCAS have been described following past influenza pandemics since the late 19th century [46,47]. Additionally, the similarities between PCAS and myalgic encephalomyelitis/chronic fatigue syndrome have been well described [48]. Do effective interventions exist, given our history with these long-term post-viral sequelae? A 2015 systematic overview of interventions for chronic fatigue syndrome found moderate certainty evidence (GRADE) for graded exercise, moclobemide, and hydrocortisone and low certainty evidence (GRADE) for cognitive behavioral therapy and selected antidepressants [49]. Similarly, for post-viral olfactory dysfunction, olfactory training has been the intervention with the most support during pre-COVID-19 times [50]. These prior studies can offer future direction for potential PCAS interventions

Next Steps

There exists room for better designed trials even for the PCAS conditions explored in this review. In this review there was a significant association with studies that did only a per protocol analysis and high risk of bias. Although an ITT analysis is not always feasible, none of the per-protocol studies reported appropriate analyses to estimate the effect of adhering to the intervention as suggested by RoB2. Researchers should consider all RoB2 domains and signaling questions when designing trials.

A meta-analysis studying post-COVID-19 populations found that over 20% experience persistent fatigue and breathlessness, while at least 10% suffer from anxiety, depression, insomnia, post-traumatic stress disorder, joint pains, and myalgia [51]. Interestingly, none of the eligible trials in our systematic review focused on populations with these mental disorders and musculoskeletal conditions. A review of registered trials looking at interventions for key mental conditions in PCAS revealed plans for 42 trials that were yet to be completed at the time of this review [52]. As time progresses, updates to this review will reveal the true extent of effective interventions for the wide spectrum of PCAS. There is also a need for PCAS trials in the pediatric age group, seeing that they are not spared from the long-term effects of COVID-19 and were underrepresented in this review.

Strengths and limitations

This systematic review had some key strengths. The comprehensiveness of the review was ensured by well-defined eligibility criteria. The inclusion criteria of trials that enrolled or randomized participants with new and ongoing symptoms at least 90 days post-acute COVID-19 infection optimized the inclusion of eligible long COVID-19 trials. The search strategy spanned multiple sources and included unpublished trials. The use of paired independent reviewers and the application of the Cochrane risk-of-bias [RoB2] tool for independent risk-of-bias assessment lent rigor to the analysis. Lastly, in summarizing all PCAS trials, this review revealed significant research gaps which can guide further research.

There are a few limitations of this review. Firstly, it was limited to English literature and may have missed articles published in other languages. Secondly, it is possible that eligible papers could have been missed, especially if they were not described as trials in their title. Thirdly, the grey literature was sourced through Google Scholar, though its coverage may not be exhaustive and there is a possibility that unpublished trial reports may have been overlooked. Lastly, PCR confirmation of COVID-19 was not explicitly stated in all studies. However, these studies' inclusion criteria required participants to not only have an initial diagnosis of COVID-19 infection but also to exhibit the development of new symptoms post-diagnosis.

## Conclusions

This systematic review found most of the trials on interventions for treating post-COVID-19 syndrome were at high bias risk. Although previous non-COVID-19 post-viral and existing post-COVID-19 syndrome trials provide some treatment leads, further robust trials and novel treatments are needed. Interventional study research gaps also exist for post-COVID-19 mental disorders, musculoskeletal issues, and pediatric patients.
